# An Innovative, Multimedia-Based Intervention for Promoting Changes in Lifestyle in Italian Families

**DOI:** 10.3390/nu18172851

**Published:** 2026-09-01

**Authors:** Iolanda Chinellato, Annamaria Acquaviva, Mary Lista, Loreto Nemi, Eva Da Ros, Benedetta Morlupi, Manuela Maione, Renata Carraro, Laura Lodi, Alessandra Micozzi, Rosa Cipriano, Patrizia Serra, Monica Turchetto, Marco Sandri, Francesca Poggiante

**Affiliations:** 1 Presidio Ospedaliero San Pio da Pietrelcina, 74011 Castellaneta, TA, Italy; iolanda.chinellato@gmail.com; 2 Istituto Oncologico Romagnolo (IOR), 47122 Forlì, FC, Italy; annamaria.acquaviva@gmail.com; 3 Independent Researcher, 00152 Rome, RM, Italy; loretonemi@gmail.com; 4 Independent Researcher, 31029 Vittorio Veneto, TV, Italy; eva.daros@gmail.com; 5 Independent Researcher, 06073 Corciano, PG, Italy; dietistamorlupi@gmail.com; 6 Ospedale del Mare, ASL Napoli 1 Centro, 80147 Napoli, NA, Italy; manuela.maione78@gmail.com; 7 Independent Researcher, 25122 Brescia, BS, Italy; renata@studiodietisticocarraro.it; 8 Independent Researcher, 41012 Carpi, MO, Italy; info@dietistalauralodi.it; 9 Faculty of Social and Communication Sciences, Universitas Mercatorum, 00186 Rome, RM, Italy; alessandra.micozzi@unimercatorum.it; 10 Independent Researcher, 80045 Pompei, NA, Italy; rosacip23@gmail.com; 11 Biostatistics and Clinical Trial Unit, Istituto Romagnolo per lo Studio dei Tumori “Dino Amadori” IRST IRCCS, 47014 Meldola, FC, Italy; patrizia.serra@irst.emr.it; 12 Independent Researcher, 20157 Milano, MI, Italy; drssa.monicaturchetto@gmail.com; 13 Big and Open Data Innovation Laboratory (BODaI-Lab), University of Brescia, 25122 Brescia, BS, Italy; 14 Independent Researcher, 80131 Napoli, NA, Italy; poggiante@dietaoggi.it

**Keywords:** childhood and parental obesity, eating habits, Mediterranean diet, health promotion, digital platforms

## Abstract

**Background/Objectives:** Several studies have reported a high prevalence of overweight and obesity among children and adults, with relevant implications for long-term health. Digital platforms and social media may support nutrition education when their content is developed and supervised by health professionals. This study evaluated whether dietary habits, lifestyle indicators, body mass index-related measures, and Mediterranean diet adherence differed between baseline and 210-day assessments among participants in a family- and school-mediated multimedia educational program aimed at promoting healthier lifestyles and greater adherence to the Mediterranean diet. **Methods:** This was an uncontrolled repeated cross-sectional before-and-after evaluation based on online surveys administered at baseline and after 210 days to respondent samples from the same project population. The multimedia educational program was delivered through a dedicated website and social media channels. The analysis included 329 children/adolescents at both assessment waves and 119 and 122 adult female respondents at baseline and 210 days, respectively. Since only 11 adult males responded, analyses of adults were restricted to women. Additionally, Mediterranean diet adherence was assessed using a modified KIDMED-derived score in children/adolescents and the PREDIMED score in adults. Data from the two assessment waves were compared at the group level, rather than as paired longitudinal observations. **Results:** Among children/adolescents, higher physical activity, lower screen time, and more favorable dietary indicators were observed at 210 days than at baseline. Moreover, daily fruit intake, daily vegetable intake, regular fish consumption, and the modified KIDMED score were higher at 210 days. Good adherence to the Mediterranean diet increased from 46.8% at baseline to 61.4% at 210 days. Among adult female respondents, the median body mass index was lower at 210 days than at baseline, and favorable differences were observed for vegetable, fish/seafood, legume, and nut intake. The median PREDIMED score increased from 7.0 [6.0–8.0] at baseline to 9.0 [7.0–10.0] at 210 days, and good Mediterranean diet adherence increased from 10.9% to 35.2%. **Conclusions:** Among children/adolescents, physical activity, screen time, and dietary indicators showed more favorable distributions in the 210-day respondent group than in the baseline respondent group. Daily fruit intake, daily vegetable intake, regular fish consumption, and the modified KIDMED-derived score were higher at 210 days, and good Mediterranean diet adherence was more frequent at 210 days than at baseline (61.4% vs. 46.8%). Among adult female respondents, the median body mass index was lower in the 210-day respondent group than in the baseline respondent group, and more favorable distributions were observed for vegetable, fish/seafood, legume, and nut intake. The median PREDIMED score was higher at 210 days than at baseline: 9.0 [7.0–10.0] versus 7.0 [6.0–8.0], respectively. Good Mediterranean diet adherence was also more frequent at 210 days (35.2% vs. 10.9%).

## 1. Introduction

In recent decades, epidemiological studies have reported poor dietary habits and unhealthy lifestyles among children and adolescents [1,2,3,4,5,6], together with an increasing prevalence of cardiovascular risk factors and eating disorders among adults [7]. Educational and lifestyle interventions have been shown to promote healthier behaviors and contribute to obesity prevention among children and adolescents, particularly when they involve family- and school-based approaches [8,9].

Childhood overweight and obesity represent a major and growing public health challenge worldwide. According to the World Health Organization, more than 390 million children and adolescents aged 5–19 years were overweight in 2022, and the prevalence of overweight in this age group increased from 8% in 1990 to 20% in 2022 [10]. Obesity during childhood and adolescence is associated with a substantial burden of disease, including cardio-metabolic, endocrine, respiratory, psychosocial, and long-term health complications [11].

In the WHO European Region, the latest Childhood Obesity Surveillance Initiative (COSI) reported that, overall, 25% of children aged 7–9 years were living with overweight (including obesity), while 11% were living with obesity, with substantial differences in prevalence across countries [12].

According to the Italian PASSI surveillance system, approximately 50% of adults in Italy are overweight or obese [13], and according to the OKkio alla Salute Childhood Obesity Italian surveillance system, approximately 30% of children in Italy are overweight or obese [1]. Thus, according to the national survey 2023 OKkio alla Salute data, Italy remains among the European countries with the highest prevalence of weight excess in its school-age population, with 19% of children classified as overweight, 9.8% as obese, including 2.6% with severe obesity, with well-documented health consequences (Italian National Institute of Health—Istituto Superiore di Sanità—OKkio alla Salute 2023 National Surveillance).

While a decline in childhood overweight and obesity was observed in Italy between 2008 and 2016 [14], excess weight remains a major public health concern.

Childhood obesity has been associated with increased psychological distress (OR = 2.44; 95% CI: 1.12–5.27), bullying victimization (OR = 2.25; 95% CI: 1.17–4.34), and a higher prevalence among children from disadvantaged socioeconomic backgrounds [1,15].

Despite many educational initiatives and pediatric recommendations, achieving a lasting change in dietary habits remains challenging. In this context, the Mediterranean diet represents a well-established nutritional model for preventing chronic degenerative diseases and promoting health-related quality of life [16,17].

Major determinants of higher adherence to the Mediterranean diet include a younger age, a higher physical activity level, adequate sleep duration, and, among dietary habits, consuming breakfast and eating with family members and at school. Parents’ younger age and higher education status were also determinants of higher adherence. Multivariate adjusted analyses showed that an overall healthier lifestyle and parents’ education were the factors independently associated with higher adherence to the Mediterranean diet [18]. A substantial body of research has shown the critical influence of the family in cultivating positive eating habits and fostering a healthy relationship with food in children through role modeling and responsive, authoritative parenting. Conversely, controlling or restrictive styles may contribute to dysfunctional eating patterns [19].

The present study was developed to test educational tools aimed at promoting healthier lifestyles among children and families and improving family dietary habits through the rediscovery of preparing and sharing daily meals, starting with breakfast. To address this objective, we designed and implemented a multimedia educational program involving more than 100 dietitian nutritionists. The program included a dedicated website with sections specifically designed for parents and other family adults, children, and teachers, as well as social media pages on Facebook and Instagram. A dedicated Facebook page for teachers was also created to provide educational materials.

These platforms allowed us to provide evidence-based information to more than 91,000 users between 2019 and 2021.

Recent studies suggest that social media platforms, if used appropriately, can be powerful tools for health education [20,21]. Thus, our project fostered ongoing dialog between families and professionals, with encouraging results, particularly among children who engaged with dietitians via social media or by participating in a recipe contest, that is, creating healthy and tasty meals together with one or both parents.

Children’s eating behaviors are strongly influenced by the family and educational environment. Parents, teachers, and grandparents play an important role in encouraging children to try new foods and acquire healthier eating habits. The BATMAN Project (Bambini A Tavola, https://www-archivio.irst.emr.it/it/irst-informa/notizie/tutte-le-notizie/notizie-2016/progetto-batman-bambini-a-tavola-20-aprile-2016.html, accessed on 7 July 2026), an observational nutrition education initiative promoted by the Istituto Oncologico Romagnolo and supported by IRST IRCCS, served as a pilot experience for the subsequent development of the “Sano, Giusto e con Gusto!” project (www.sanogiustoecongusto.it, accessed on 7 July 2026). Observations emerging from that preliminary experience contributed to the design of the present educational intervention, particularly regarding family involvement, food tasting activities, and participation in meal preparation. The BATMAN Project suggested that tasting experiences and active participation in meal preparation may improve children’s taste preferences.

Additionally, the use of simple recipes and cooking as a playful educational activity may also promote food awareness and the adoption of healthier lifestyle habits. Building on these premises, “Sano, Giusto e con Gusto!” was designed to extend this educational approach from a local setting to a national setting, involving children and families in preparing healthy recipes through a national contest.

The objective of this study was to evaluate whether dietary habits, lifestyle indicators, BMI-related measures, and Mediterranean diet adherence differed between baseline and 210-day assessments among adults and children/adolescents participating in the “Sano, Giusto e con Gusto!” multimedia educational program. As the quality of online health information is often variable, professionally developed and supervised educational content has been recommended to improve the reliability and effectiveness of digital nutrition interventions [22,23,24,25,26,27,28].

## 2. Materials and Methods

This section describes the study design, participant recruitment strategy, eligibility criteria, intervention components, data collection procedures, measurement instruments, and study variables used to evaluate the multimedia educational program.

### 2.1. Study Design and Setting

This was an uncontrolled pre–post evaluation based on repeated cross-sectional surveys. Thus, data were collected before and after a multimedia educational intervention. Moreover, since participants responding at baseline and after the intervention were not necessarily the same individuals, this study was not designed as a paired longitudinal cohort, and individual-level changes over time could not be estimated. No randomization was performed, and no parallel control group was included.

The study covered one school year. This duration was chosen to allow baseline assessment, intervention delivery, and post-intervention assessment within the same school year; that is, before final-year primary school and lower secondary school classes changed teachers and therefore lost their reference adult within the project.

### 2.2. Participants and Recruitment

The study included two analytical groups: female adults and children/adolescents. Participants were recruited from users registered for the project and contacted through the project newsletter. The invitation was sent to all registered users, specifying that individuals who had already been exposed to the educational program during previous editions or had previously participated in the “Healthy Plate” recipe contest were not eligible to reduce potential bias due to prior knowledge of the intervention. Accordingly, the study was based on voluntary participation among first-time registrants of the 2021–2022 project edition.

The female adult group included adult women who participated as parents, teachers, educators, grandparents, or other adults involved in guiding children/adolescents during the educational activities. Adult participants were included after accepting the invitation, providing consent, and completing the adult questionnaire. Conversely, adult males were not included in the present analytical sample because their number was too small to provide stable estimates.

The group of children/adolescents included participants who were involved in the project through a reference adult. They were either children or grandchildren of participating parents, guardians, or grandparents, or pupils from school classes whose teachers joined the project. The questionnaire link for children/adolescents was sent to the participating adults, who forwarded it to the children/adolescents and assisted younger participants when needed. The questionnaires for children/adolescents were identified using an invented nickname followed by a number.

The adult and child/adolescent samples should not be interpreted as matched adult–child/adolescent pairs. Rather, children/adolescents were connected to the project through participating adults, but the analyses were conducted at the group level.

### 2.3. Eligibility Criteria

Participants were eligible if they were first-time participants in the 2021–2022 edition of the project and had not previously been exposed to the educational materials or activities of the program. Individuals who had already completed the educational pathway, viewed the educational materials, or taken part in previous project activities, including the “Healthy Plate” recipe contest, were not eligible because prior exposure could have influenced their responses to the questionnaire.

For adults, eligible participants were adult project registrants involved as parents, teachers, educators, grandparents, or other reference adults. Conversely, for children/adolescents, eligible participants were those involved in the project through a participating reference adult, either as children of the participating parents or guardians or as pupils from school classes whose teachers participated in the project.

Duplicate adult registrations were removed using the email address as the reference identifier.

### 2.4. Intervention

**Figure 1:**
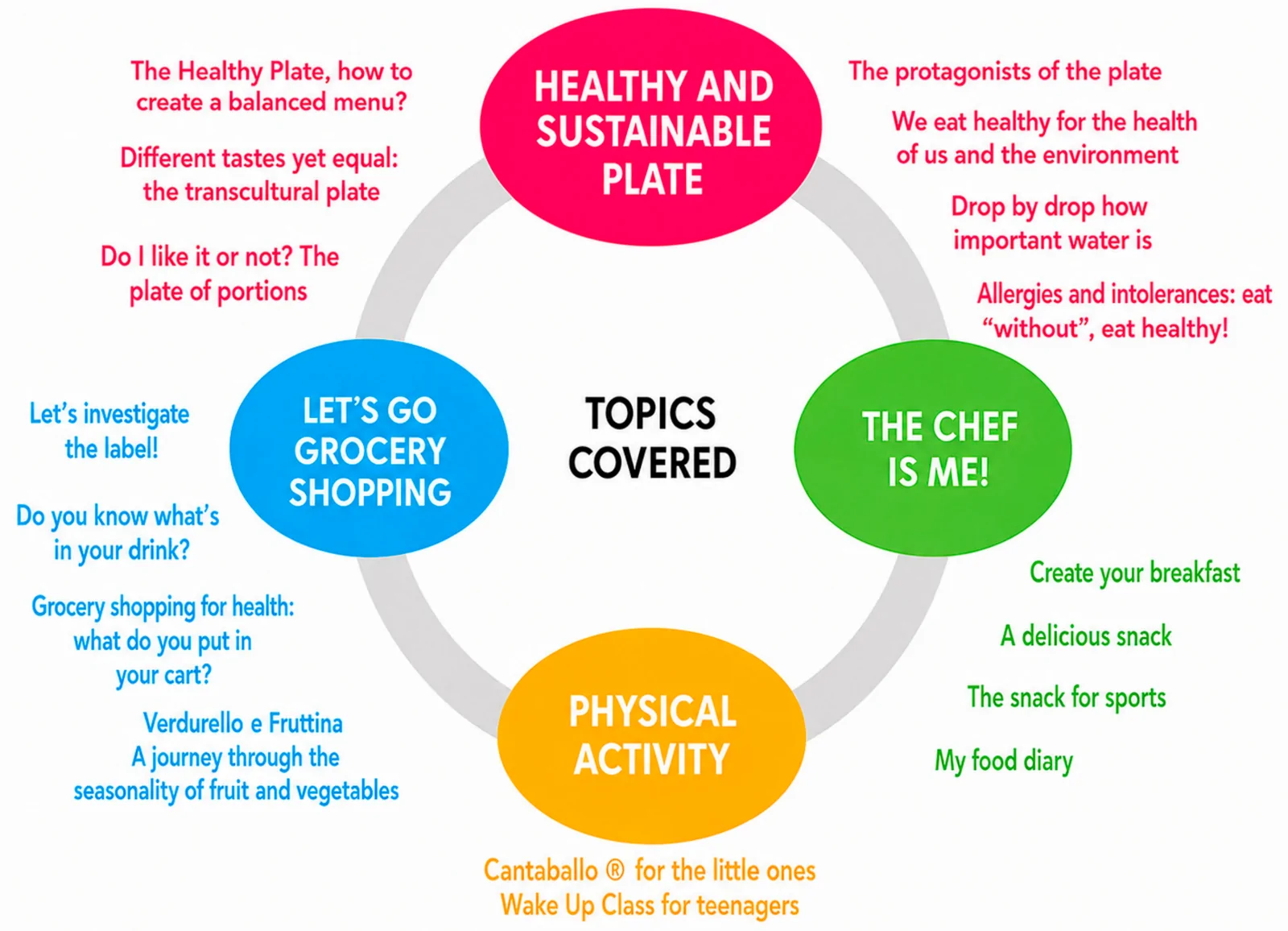
Guidance materials, worksheets, and short videos.

The intervention consisted of a family-oriented multimedia educational program delivered through the project website and social media channels. The program was structured to provide adults with evidence-based educational content and practical resources that could be used in family- or school-mediated activities with children and adolescents. The intervention also included materials directly addressed to children and adolescents, with the aim of reinforcing nutrition education through interactive and practical activities. The main components of the intervention are summarized in Figure 1.

The communication infrastructure of the project included a dedicated website, a Facebook page, a Facebook group reserved for teachers, and an Instagram page. The content was developed with the voluntary contribution of dietitian nutritionists involved in the project, together with a motor activity educator who developed the physical activity component. The website also included a section listing dietitian nutritionists by geographical area, which was intended to provide a reference point for registered participants.

The project was introduced through an online event organized into eight modules. These modules presented the scientific background of the project, its objectives and target population, participation procedures, and instructions for using the educational materials. The modules were released according to a scheduled program on the project Facebook page, “Sano, Giusto e con Gusto,” and were subsequently shared through the social media channels of the professionals involved in the project. The recordings remained available in the events section of the project website, and the eight modules covered the following topics: scientific foundations of the project; project objectives, target population, and implementation procedures; adolescents and food; the educational pathway; food intolerances, cross-cultural plates, and environmental sustainability; physical activity; cooking together; and registration procedures for the project and the recipe contest.

The adult-oriented component included downloadable educational materials, worksheets, short videos, and practical guidance on healthy eating. These materials were intended to support adults in improving their own knowledge and dietary habits and in transferring appropriate nutrition messages to children and adolescents during family- or school-based activities.

In contrast, the child/adolescent-oriented component included interactive downloadable resources, such as games, quizzes, crosswords, riddles, and other educational activities focused on nutrition. The website also included storytelling materials and video fairy tales on food-related topics that were specifically intended for younger children. Physical activity resources consisted of educational sheets and videos developed by a motor activity expert using the “Cantaballo” method.

A practical component of the intervention was the “Healthy Plate” recipe contest, which encouraged adults and children/adolescents to prepare healthy recipes together. The contest was intended to promote active participation, food tasting, and shared meal preparation, using cooking as a practical tool to reinforce the program’s educational content.

The food education intervention was delivered from November to the end of March, and its overall aim was to provide multimedia educational content and practical resources that could be used by adults, families, and schools to promote Mediterranean diet adherence and healthier lifestyle habits among children and adolescents.

### 2.5. Data Collection

Data were collected using online questionnaires administered through Google Forms (Google LLC, Mountain View, CA, USA). Data collection was conducted at two time points: before the educational intervention and after completion of the intervention period.

Adult dietary habits were assessed using the validated PREDIMED questionnaire, which measures adherence to the Mediterranean diet [29]. For this study, the questionnaire was supplemented with items on the participants’ role in the project, educational level, age, region of residence, body weight, and height. The questionnaire link was sent by email to adult participants. Before completing the questionnaire, participants were required to accept the privacy information and provide consent to participate, and duplicate project registrations were removed using the email address as the reference identifier. However, repeated email addresses in questionnaire responses were not automatically treated as duplicates, as the same email address could be used by more than one adult family member or reference adult. In these cases, records were reviewed using the available questionnaire information, and only true duplicate submissions by the same respondent were excluded.

Dietary habits in children/adolescents were assessed using a questionnaire adapted from the validated KIDMED instrument, which measures adherence to the Mediterranean diet in children and adolescents [30]. The first KIDMED item was modified by replacing fruit juices with freshly squeezed juice or blended fruit. Additional items collected information on body weight, height, age, region of residence, physical activity, leisure screen time, and the reference adult involved in the project.

The questionnaire link for children/adolescents was sent to the participating reference adults, who forwarded it to the children/adolescents and assisted them when needed. Children/adolescents questionnaires were identified using an invented nickname followed by a number. Additionally, consent documentation and photo/video release forms were attached to the study invitation.

The baseline data collection period lasted two months and was conducted at the beginning of the school year. After the educational intervention, the questionnaires were administered again using the same procedures: adults completed the modified PREDIMED questionnaire, while children/adolescents completed the modified KIDMED questionnaire.

### 2.6. Measurement Instruments and Score Calculation

Mediterranean diet adherence in adults was evaluated using the modified PREDIMED score. The questionnaire included 14 items, and one point was assigned to each answer indicating adherence to the Mediterranean diet. The total score ranged from 0 to 14, and adherence was classified as low for scores of 0–5, moderate for scores of 6–9, and good for scores of 10 or higher.

In contrast, adherence in children/adolescents was evaluated using a modified KIDMED-derived score. The original KIDMED questionnaire includes 16 items. In this study, two additional items were included: one on physical activity and one on leisure screen time. Positive adherence items contributed +1 point, whereas counter-adherence items contributed −1 point. The total score ranged from 0 to 13, and adherence was classified as poor for scores ≤ 3, moderate for scores of 4–7, and good for scores ≥ 8. Because the original KIDMED instrument was modified, these categories were used for descriptive purposes and should not be interpreted as externally validated cutoffs for the modified score.

### 2.7. Study Variables

The evaluation focused on variables related to nutritional status, dietary habits, Mediterranean diet adherence, and lifestyle behaviors.

In adults, the main variables included body mass index, individual PREDIMED items, the PREDIMED score, and the corresponding Mediterranean diet adherence category. Additional adult variables included age, sex, geographical area, educational level, and respondent role.

In children/adolescents, the main variables included BMI, BMI category, individual KIDMED items, the modified KIDMED score, and the corresponding Mediterranean diet adherence category. BMI categories were assigned using the Cole/IOTF age- and sex-specific cutoffs, which are recommended for pediatric populations because BMI interpretation varies according to age and sex [31]. Additional variables included age, sex, geographical area, physical activity, leisure screen time, and the reference adult involved in the project.

Educational level was recorded in adults and was considered a descriptive and stratification variable for evaluating differences in Mediterranean diet adherence across educational groups.

### 2.8. Statistical Analysis

Adults and children/adolescents were analyzed separately. Within each population, baseline and 210-day respondents were considered as independent samples, as the two assessments included only partially overlapping participants rather than the same individuals measured at both time points. Therefore, comparisons between baseline and 210 days were performed using statistical methods for independent samples.

Categorical variables were summarized as absolute frequencies and percentages and were compared between baseline and 210-day respondents using Fisher’s exact test. Continuous and discrete score variables were summarized as medians and interquartile ranges and compared between baseline and 210-day respondents using the Kruskal–Wallis rank test.

In addition to unadjusted comparisons, adjusted analyses of questionnaire score distributions were performed using quantile regression. The assessment time point was included as the main independent variable to estimate adjusted differences between baseline and 210-day respondents, and models were fitted separately for female adults and children/adolescents. In adults, models were adjusted for age, educational level, and geographical area of residence. Conversely, they were adjusted for sex, age, and geographical area of residence in children/adolescents.

No formal a priori sample-size calculation was performed, as the study was embedded in an ongoing educational project and the analytical sample was determined by the number of eligible respondents who completed the questionnaires at baseline and after 210 days. We therefore calculated the minimum difference in questionnaire scores that could be detected with the achieved sample sizes. Using PASS 2021, version 21.0.4 (NCSS, LLC, Kaysville, UT, USA), and assuming a two-sided Wilcoxon rank-sum test, α = 0.05, 80% power, an interquartile range width of 5 points, and a reference median value of 7 points, the available samples had 80% power to detect differences of approximately 0.89 KIDMED points in children/adolescents and 1.47 PREDIMED points in adults. Under these assumptions, the achieved sample sizes had sufficient statistical sensitivity to detect score differences of the magnitude observed in this study. However, this post hoc sensitivity analysis should not be interpreted as a substitute for an a priori sample-size calculation.

Because the study was uncontrolled and based on voluntary participation, statistical comparisons were interpreted as exploratory and descriptive of observed group-level changes rather than as evidence of individual changes or causal intervention effects.

Given the exploratory nature of the study, no adjustment for multiple comparisons was applied; therefore, *p*-values should be interpreted descriptively and not as confirmatory evidence.

A two-sided *p*-value < 0.05 was considered statistically significant, and all analyses were performed using Stata Statistical Software: Release 18 (StataCorp 2023, College Station, TX, USA).

## 3. Results

### 3.1. Children and Adolescents

Table 1 reports anthropometric characteristics, lifestyle indicators, selected dietary habits, and Mediterranean diet adherence among children and adolescents at baseline and after 210 days. The analysis included 329 respondents at T0 and 329 respondents at T210. At baseline, 149 participants were male, while 180 were female; the sex distribution did not differ significantly between T0 and T210 (*p* = 0.39). However, the distribution of the reference adult involved in the child/adolescent’s participation differed between time points, with teacher/instructor involvement increasing from 35.6% to 54.7% (*p* < 0.001).

For BMI classification, children/adolescents were analyzed in two age groups: 6–10 years and 11–14 years. Among children aged 6–10 years, the distribution of BMI categories did not differ significantly between T0 and T210 (*p* = 0.778). At T0, 9% were underweight, 22% were mildly underweight, 14% had a normal weight, 38% were overweight, and 17% had obesity. At T210, the corresponding percentages were 9%, 20%, 18%, 37%, and 16%, respectively. Among children/adolescents aged 11–14 years, the BMI category distribution also did not differ significantly between T0 and T210 (*p* = 0.211). At T0, 7% were underweight, 29% were mildly underweight, 8% had a normal weight, 47% were overweight, and 9% had obesity; in contrast, the corresponding percentages were 11%, 29%, 19%, 32%, and 9%, respectively, at T210.

Physical activity was more favorable in the T210 respondent group than in the T0 respondent group (*p* = 0.023). The proportion reporting physical activity two to three times per week was higher at T210 than at T0 (64.7% vs. 55.6%), whereas the proportion reporting no physical activity was lower (4.0% vs. 7.6%). Additionally, screen time also showed a more favorable distribution at T210 (*p* < 0.001): the proportion spending more than 3 h per day watching TV or playing video games was lower at T210 than at T0 (9.1% vs. 18.8%), whereas the proportion reporting no more than 1 h per day was higher (45.9% vs. 31.3%).

Several dietary indicators showed more favorable distributions in the T210 respondent group than in the T0 respondent group. The proportion reporting daily fruit intake was higher at T210 than at T0 (84.2% vs. 72.3%; *p* < 0.001), as was the proportion reporting a second daily serving of fruit (63.8% vs. 40.4%; *p* < 0.001). Additionally, daily vegetable intake was also more frequent at T210 (78.4% vs. 60.5%; *p* < 0.001), and the proportion reporting vegetables more than once per day was higher (58.1% vs. 31.6%; *p* < 0.001). Lastly, regular fish consumption was more frequently reported at T210 than at T0 (57.8% vs. 45.0%; *p* = 0.001).

The median modified KIDMED-derived score was higher in the T210 respondent group than in the T0 respondent group: 8.0 [6.0–11.0] versus 7.0 [5.0–9.0], respectively (*p* < 0.001). Mediterranean diet adherence categories also showed a more favorable distribution at T210 (*p* < 0.001): good adherence was more frequent at T210 than at T0 (61.4% vs. 46.8%), whereas moderate adherence (35.9% vs. 43.5%) and poor adherence (2.7% vs. 9.7%) were less frequent. In the multivariable median regression model, the modified KIDMED score was compared between T0 and T210 after adjusting for sex, age, and geographical area. The adjusted median score was higher at T210 than at T0, with an estimated difference of 1.50 points (95% CI, 0.98 to 2.02; *p* < 0.001). This result indicates that the difference observed at T210 was retained after adjusting for the main demographic characteristics of the child/adolescent sample.

### 3.2. Adult Respondents

Table 2 reports body mass index, selected dietary habits, and Mediterranean diet adherence among adult respondents at baseline and after 210 days. The analysis included 119 respondents at T0 and 122 respondents at T210, with median BMI lower in the T210 respondent group than in the T0 respondent group: 23.4 kg/m^2^ [21.3–27.0] versus 24.7 kg/m^2^ [22.1–28.1], respectively (*p* = 0.014). The distribution of BMI categories showed a higher proportion of normal-weight respondents at T210 than at T0 (64.0% vs. 54.6%) and a lower proportion with obesity (7.2% vs. 17.6%), although the overall comparison of BMI categories did not reach statistical significance (*p* = 0.059).

Selected dietary indicators showed more favorable distributions in the T210 respondent group than in the T0 respondent group. The proportion reporting at least two servings of vegetables per day was higher at T210 than at T0 (55.7% vs. 36.1%; *p* = 0.002). Additionally, weekly fish/seafood intake of at least three servings was more frequently reported at T210 (15.6% vs. 3.4%; *p* = 0.003), as was weekly legume intake of at least three servings (17.2% vs. 6.7%; *p* = 0.044).

The median PREDIMED score was higher in the T210 respondent group than in the T0 respondent group: 9.0 [7.0–10.0] versus 7.0 [6.0–8.0], respectively (*p* < 0.001). Mediterranean diet adherence categories also showed a more favorable distribution at T210 (*p* < 0.001): good adherence was more frequent at T210 than at T0 (35.2% vs. 10.9%), whereas moderate adherence (62.3% vs. 71.4%) and low adherence (2.5% vs. 17.6%) were less frequent. After adjustment for age, geographical area, and educational level, the PREDIMED score remained higher at T210 than at T0 (adjusted median difference: two points; 95% CI, 1.4 to 2.6; *p* < 0.001).

### 3.3. Secondary Outcomes

As an exploratory secondary analysis, educational level was grouped into primary/lower secondary education and upper secondary education/university degree, and obesity prevalence was compared between baseline and 210 days within each stratum. Among respondents with upper secondary education or a university degree, obesity was less frequent at 210 days than at baseline, decreasing from 16.7% to 3.2%. This difference remained statistically significant after adjusting for age and geographical area in a multivariable logistic regression model with obesity as the dependent variable (odds ratio: 0.23, 95% CI, 0.04–0.87; *p* = 0.026). By contrast, no comparable reduction was observed among respondents with primary or lower secondary education, in whom obesity was 20.8% at baseline and 29.4% at 210 days.

## 4. Discussion

This exploratory uncontrolled repeated cross-sectional evaluation showed favorable group-level differences in Mediterranean diet adherence, selected dietary habits, physical activity indicators, and screen-time behaviors between baseline and 210-day assessments among participants involved in a family- and school-mediated multimedia educational program. However, because this was a non-randomized, uncontrolled evaluation without paired follow-up of the same individuals, these findings should be interpreted as descriptive observations and hypothesis-generating results rather than as evidence of within-person changes or causal effects attributable to the intervention.

The observations regarding Mediterranean diet adherence are consistent with a growing body of literature suggesting that family-based nutrition education programs and digitally delivered educational initiatives may support healthier dietary behaviors [16,32,33]. Additionally, previous studies have highlighted the importance of involving both families and educational settings in promoting adherence to the Mediterranean diet and improving nutrition-related knowledge and behaviors among children and adolescents [16,32].

The Mediterranean diet remains one of the most extensively studied dietary patterns and has been associated with favorable health outcomes in children, adolescents, and adults [30]. Therefore, the favorable differences observed in both children/adolescents and adult respondents may be considered encouraging from a public health perspective, although future controlled longitudinal studies are needed to determine whether and to what extent these observations can be attributed to the educational program itself.

Beyond dietary indicators, the study also found favorable differences in lifestyle-related variables, including physical activity and recreational screen exposure among children and adolescents. These findings are relevant because sedentary behaviors and excessive screen time have been associated with poorer diet quality, lower levels of physical activity, and an increased risk of overweight and obesity in pediatric populations [34,35]. The simultaneous observation of dietary and lifestyle indicators may support the value of integrated educational approaches that address multiple health-related behaviors rather than focusing exclusively on nutrition.

Another relevant aspect concerns the use of digital platforms and social media as channels for nutrition education. The rapid growth of digital communication has increased access to health-related information, although concerns remain regarding the quality, reliability, and scientific accuracy of online content. Recent evidence suggests that professionally designed and supervised digital interventions may improve nutrition knowledge, engagement, and healthy eating behaviors, particularly among younger populations [26,36]. A distinctive feature of the present project was that educational materials were primarily mediated by parents, teachers, and educators rather than being directly delivered to children, thereby integrating digital resources within family and school environments. Additionally, in the present project, educational materials were developed and reviewed by health professionals and delivered through a structured multimedia pathway involving families, schools, and healthcare professionals. The findings of this exploratory evaluation warrant further investigation into professionally supervised digital platforms as complementary tools for nutrition education and health promotion.

The exploratory secondary analysis suggested that educational level may be associated with differences in obesity prevalence among adult respondents, and similar associations between health literacy, food literacy, dietary quality, and health-related behaviors have been described in previous studies [37]. Although the present findings should be interpreted cautiously, they suggest the potential importance of educational and social determinants when designing future nutrition education initiatives.

These observations are consistent with the broader public health perspective that health behaviors are influenced not only by individual choices but also by educational, social, and environmental factors.

The strengths of this study include the nationwide implementation of the project, the involvement of both adults and children/adolescents, and the integration of family, school, and healthcare settings within a single educational framework. In addition, the program combined evidence-based educational materials with practical activities, interactive resources, educational games, social media communication, and family-oriented experiences, reflecting contemporary approaches to health promotion. Additionally, the multidisciplinary collaboration among healthcare professionals, educators, and families represents a further strength of the project and may have contributed to participant engagement throughout the intervention period.

This study has some important methodological limitations that should be considered when interpreting the findings.

The main limitation is that the baseline and 210-day samples were only partially overlapping. All participants assessed at T210 had been exposed to the educational intervention, but they were not necessarily the same individuals assessed at T0. This applies to both adult respondents and children/adolescents. Therefore, the T0–T210 comparison cannot be interpreted as a longitudinal within-person comparison. Thus, the findings describe group-level differences between two assessment periods, rather than individual changes in the same participants over time.

This study also lacked a parallel control group, and no randomization was performed. Consequently, the observed differences between T0 and T210 cannot be attributed with certainty to the educational intervention, as other unmeasured factors occurring during the same period may have contributed to the findings.

The sample was not randomly selected but was based on voluntary participation among project registrants who responded to the newsletter invitation. Selection bias cannot be excluded, and the findings should not be interpreted as representative of the general population. In addition, the adult analytical sample included only female respondents; therefore, the adult findings cannot be generalized to men, and sex-specific differences in dietary habits, BMI, or Mediterranean diet adherence could not be evaluated in adults.

The composition of the samples differed between T0 and T210 for some characteristics, including geographical area among adults and reference adult among children/adolescents. Although adjusted analyses were performed, residual confounding due to measured and unmeasured differences between respondents at the two assessment periods cannot be excluded.

All data were collected through online self-administered questionnaires, and weight, height, dietary habits, physical activity, and screen time were self-reported or reported with adult support for younger participants. These measures may be affected by recall bias, reporting bias, and social desirability bias. Additionally, self-reported anthropometric data may also have introduced measurement errors in BMI estimation and BMI category classification.

Finally, the intervention included several digital and educational components, including website materials, social media content, educational worksheets, videos, games, and the recipe contest. However, individual-level exposure to these components was not measured. Therefore, the relationship between the intensity of exposure to the intervention materials and changes in questionnaire scores could not be assessed.

The follow-up period was also limited to one school year, preventing evaluation of the long-term maintenance of dietary habits, physical activity, screen-time behaviors, or Mediterranean diet adherence.

Despite these limitations, the present exploratory findings indicate that a family- and school-mediated multimedia educational approach is feasible and may be associated with more favorable dietary and lifestyle profiles. Thus, future controlled longitudinal studies with repeated measurements in the same participants and longer follow-up periods are needed to assess the reproducibility and long-term sustainability of these observations.

## 5. Conclusions

Several studies have shown that social media may represent a useful tool for health promotion, facilitating the creation of user networks and providing healthcare institutions and professionals with a stable communication channel for interacting with families and the public [16,22]. When appropriately managed, digital platforms may support the dissemination of evidence-based information and promote interaction with the public, thereby facilitating engagement in prevention programs.

In the present study, the educational intervention combined social media pages, including Facebook, Instagram, and a channel dedicated to teachers, with an interactive website specifically developed to provide educational content, downloadable teaching materials, and self-monitoring tools for adults and children/adolescents. This multichannel structure was intended to provide accessible and flexible educational content tailored to different age groups and educational contexts.

After 210 days, favorable group-level differences were observed in several dietary and lifestyle indicators. In children and adolescents, these differences included a higher adherence to the Mediterranean diet, lower screen time, and a higher frequency of physical activity. In adult respondents, higher PREDIMED scores and a greater proportion of respondents with good Mediterranean diet adherence were observed at 210 days than at baseline. Additionally, modest favorable differences in BMI and BMI category distribution were also observed, although these findings should be interpreted cautiously because of the uncontrolled design and the partial overlap between respondent groups assessed at baseline and after 210 days.

Moreover, the exploratory secondary analysis suggested that the reduction in obesity prevalence between baseline and 210 days was mainly limited to respondents with upper secondary education or a university degree. Although this association remained significant after adjusting for age and geographical area, future studies should examine whether regional differences across Italy may contribute to these patterns.

In summary, these preliminary findings suggest that an integrated, family-based digital educational program may represent a promising approach to promoting Mediterranean diet adherence and healthier lifestyle behaviors in children, adolescents, and adults. The involvement of parents, teachers, and health professionals may also be relevant for supporting engagement with educational activities. However, the absence of a control group, lack of randomization, the use of self-reported data, a partial overlap between respondent groups, and a relatively short observation period limit causal interpretation. Future controlled studies with individual-level follow-up and longer observation periods are needed to confirm these findings and to assess whether such interventions can produce sustained individual-level changes in dietary habits, lifestyle indicators, and body weight.

## Figures and Tables

**Table 1:** Anthropometric characteristics, lifestyle indicators, dietary habits, and Mediterranean diet adherence among children and adolescents at baseline and after 210 days.

	Timepoint		
	T0	T210	*p*-Value
N	329 (50.0%)	329 (50.0%)	
Sex			
Male	149 (45.3%)	161 (48.9%)	0.390
Female	180 (54.7%)	168 (51.1%)	
Age (years)	10.0 [8.0–10.0]	9.0 [7.0–10.0]	0.108
Geographical area			
North	55 (16.7%)	67 (20.4%)	0.470
Centre	145 (44.1%)	135 (41.0%)	
South	129 (39.2%)	127 (38.6%)	
Reference adult			
Teacher/instructor	117 (35.6%)	180 (54.7%)	<0.001
Parent/guardian	86 (26.1%)	55 (16.7%)	
Both	126 (38.3%)	94 (28.6%)	
Body mass index (kg/m^2^)	17.7 [15.8–20.5]	17.3 [15.8–20.1]	0.257
BMI for children aged 6–10 years			
Underweight	23 (9%)	23 (9%)	0.778
Mild underweight	58 (22%)	54 (20%)	
Normal weight	36 (14%)	47 (18%)	
Overweight	101 (38%)	97 (37%)	
Obesity	45 (17%)	42 (16%)	
BMI for children aged 11–14 years			
Underweight	5 (7%)	7 (11%)	0.211
Mild underweight	19 (29%)	19 (29%)	
Normal weight	5 (8%)	13 (19%)	
Overweight	31 (47%)	21 (32%)	
Obesity	6 (9%)	6 (9%)	
Physical activity			
Never	25 (7.6%)	13 (4.0%)	0.023
Once a week	121 (36.8%)	103 (31.3%)	
Two to three times a week	183 (55.6%)	213 (64.7%)	
TV/video games			
More than 3 h per day	62 (18.8%)	30 (9.1%)	<0.001
2–3 h per day	164 (49.8%)	148 (45.0%)	
0 or at most 1 h per day	103 (31.3%)	151 (45.9%)	
Fruit daily			
No	91 (27.7%)	52 (15.8%)	<0.001
Yes	238 (72.3%)	277 (84.2%)	
Second fruit daily			
No	196 (59.6%)	119 (36.2%)	<0.001
Yes	133 (40.4%)	210 (63.8%)	
Vegetables daily			
No	130 (39.5%)	71 (21.6%)	<0.001
Yes	199 (60.5%)	258 (78.4%)	
Vegetables >1/day			
No	225 (68.4%)	138 (41.9%)	<0.001
Yes	104 (31.6%)	191 (58.1%)	
Fish regularly			
No	181 (55.0%)	139 (42.2%)	0.001
Yes	148 (45.0%)	190 (57.8%)	
Fast food > 1/week			
No	297 (90.3%)	305 (92.7%)	0.328
Yes	32 (9.7%)	24 (7.3%)	
Legumes >1/week			
No	102 (31.0%)	80 (24.3%)	0.067
Yes	227 (69.0%)	249 (75.7%)	
Pasta/rice daily			
No	36 (10.9%)	36 (10.9%)	1.000
Yes	293 (89.1%)	293 (89.1%)	
Cereals at breakfast			
No	139 (42.2%)	106 (32.2%)	0.010
Yes	190 (57.8%)	223 (67.8%)	
Nuts regularly			
No	202 (61.4%)	179 (54.4%)	0.082
Yes	127 (38.6%)	150 (45.6%)	
Extra-virgin olive oil			
No	18 (5.5%)	20 (6.1%)	0.868
Yes	311 (94.5%)	309 (93.9%)	
Milk/dairy at breakfast			
No	100 (30.4%)	57 (17.3%)	<0.001
Yes	229 (69.6%)	272 (82.7%)	
Pastries at breakfast			
No	156 (47.4%)	164 (49.8%)	0.585
Yes	173 (52.6%)	165 (50.2%)	
Yogurt/cheese daily			
No	211 (64.1%)	153 (46.5%)	<0.001
Yes	118 (35.9%)	176 (53.5%)	
Sweets several/day			
No	216 (65.7%)	256 (77.8%)	<0.001
Yes	113 (34.3%)	73 (22.2%)	
Sugar-sweetened drinks			
No	250 (76.0%)	266 (80.9%)	0.155
Yes	79 (24.0%)	63 (19.1%)	
KIDMED score	7.0 [5.0–9.0]	8.0 [6.0–11.0]	<0.001
Mediterranean diet adherence			
Poor adherence	32 (9.7%)	9 (2.7%)	<0.001
Moderate adherence	143 (43.5%)	118 (35.9%)	
Good adherence	154 (46.8%)	202 (61.4%)	

Continuous variables are reported as median [IQR]. Categorical variables are reported as *n* (%). *p*-values: Wilcoxon rank-sum test for continuous and score variables; Fisher′s exact test for categorical variables. “Reference adult” indicates whether the child/adolescent was guided by a teacher/instructor, a parent/guardian, or both; T0, baseline assessment; T210, 210-day assessment; BMI, body mass index; KIDMED, Mediterranean diet quality index for children and adolescents.

**Table 2:** Characteristics, dietary habits, body mass index, and Mediterranean diet adherence among adult respondents at baseline and after 210 days.

	Timepoint		
	T0 (*n* = 119)	T210 (*n* = 122)	*p*-Value
Age (years)	43.0 [40.0–47.0]	44.0 [39.0–47.0]	0.582
Geographical area			
North	6 (5.0%)	24 (19.7%)	<0.001
Centre	0 (0.0%)	6 (4.9%)	
South	113 (95.0%)	92 (75.4%)	
Educational level			
Primary school	9 (7.6%)	4 (3.3%)	0.323
Lower secondary	17 (14.3%)	13 (10.7%)	
Upper secondary	59 (49.6%)	71 (58.2%)	
University degree	34 (28.6%)	34 (27.9%)	
Respondent’s relationship to the child/adolescent			
Teacher/instructor	9 (7.6%)	12 (9.8%)	0.649
Parent/guardian	110 (92.4%)	110 (90.2%)	
Body mass index (kg/m^2^)	24.7 [22.1–28.1]	23.4 [21.3–27.0]	0.014
Normal weight	59 (54.6%)	71 (64.0%)	0.059
Overweight	30 (27.8%)	32 (28.8%)	
Obesity	19 (17.6%)	8 (7.2%)	
Main cooking fat: extra-virgin olive oil			
Other fats incl. olive oil	6 (5.0%)	2 (1.6%)	0.168
Mainly olive oil	113 (95.0%)	120 (98.4%)	
Daily olive oil intake			
No daily use	4 (3.4%)	2 (1.6%)	0.166
<4 tbsp/day	64 (53.8%)	54 (44.3%)	
≥4 tbsp/day	51 (42.9%)	66 (54.1%)	
Daily butter/margarine intake			
Not daily	117 (98.3%)	114 (94.2%)	0.172
Daily	2 (1.7%)	7 (5.8%)	
Daily vegetable intake			
Not daily	28 (23.5%)	12 (9.8%)	0.002
≤1 serving/day	48 (40.3%)	42 (34.4%)	
≥2 servings/day	43 (36.1%)	68 (55.7%)	
Daily fruit intake			
Not daily	21 (17.6%)	13 (10.7%)	0.153
1–2 servings/day	72 (60.5%)	72 (59.0%)	
≥3 servings/day	26 (21.8%)	37 (30.3%)	
Weekly red/processed meat intake			
0–2 servings/week	65 (54.6%)	107 (87.7%)	<0.001
3–5 servings/week	47 (39.5%)	13 (10.7%)	
>5 servings/week	7 (5.9%)	2 (1.6%)	
Daily sugar-sweetened beverage intake			
Not daily	106 (89.1%)	111 (98.2%)	<0.001
Once daily	12 (10.1%)	0 (0.0%)	
Several times/day	1 (0.8%)	2 (1.8%)	
Weekly wine intake			
None or ≤7/week	111 (93.3%)	115 (94.3%)	0.891
7–14/week	7 (5.9%)	7 (5.7%)	
>14/week	1 (0.8%)	0 (0.0%)	
Weekly legume intake			
None or ≤1/week	35 (29.4%)	31 (25.4%)	0.044
2–3/week	76 (63.9%)	70 (57.4%)	
≥3/week	8 (6.7%)	21 (17.2%)	
Weekly fish/seafood intake			
None or ≤1/week	55 (46.2%)	42 (34.4%)	0.003
2–3/week	60 (50.4%)	61 (50.0%)	
≥3/week	4 (3.4%)	19 (15.6%)	
Weekly nut intake			
≤1/week	77 (64.7%)	57 (46.7%)	0.014
1–2/week	22 (18.5%)	39 (32.0%)	
≥3/week	20 (16.8%)	26 (21.3%)	
Weekly tomato sauce intake			
Never	5 (4.2%)	0 (0.0%)	<0.001
1–2/week	81 (68.1%)	42 (37.2%)	
≥2/week	33 (27.7%)	71 (62.8%)	
Weekly packaged sweets intake			
Never or ≤2/week	70 (58.8%)	94 (77.0%)	0.010
3–4/week	38 (31.9%)	21 (17.2%)	
>1/day	11 (9.2%)	7 (5.7%)	
Prefers white meat			
Prefers other meat	40 (33.6%)	14 (11.5%)	<0.001
No meat/prefers white meat	79 (66.4%)	108 (88.5%)	
PREDIMED score	7.0 [6.0–8.0]	9.0 [7.0–10.0]	<0.001
PREDIMED adherence category			
Low adherence	21 (17.6%)	3 (2.5%)	<0.001
Moderate adherence	85 (71.4%)	76 (62.3%)	
Good adherence	13 (10.9%)	43 (35.2%)	

Continuous variables are reported as median [IQR]. Categorical variables are reported as *n* (%). *p*-values: Wilcoxon rank-sum test for continuous and score variables; Fisher’s exact test for categorical variables. T0, baseline assessment; T210, 210-day assessment; BMI, body mass index; PREDIMED, Prevención con Dieta Mediterránea.

## Data Availability

The data presented in this study are available upon reasonable request from the corresponding author, subject to necessary institutional approvals.
